# Mechanisms of *Aletris spicata* (Thunb.) Franch. Extract in Asthma Therapy: Oxidative Stress, Inflammation, and Gut Microbiota

**DOI:** 10.3390/biology14060731

**Published:** 2025-06-19

**Authors:** Jing Yang, Zhiyong Chen, Yue Zhu, Teng Chen, Ying Zhou, Zuhua Wang

**Affiliations:** 1 College of Pharmaceutical Sciences, Guizhou University of Traditional Chinese Medicine, Guiyang 550025, China; yangjingqing1124@163.com (J.Y.); chenzy20233@163.com (Z.C.); moonzy0210@163.com (Y.Z.); chenteng0907@163.com (T.C.); 2 Guizhou Key Laboratory of Modern Traditional Chinese Medicine Creation, Guiyang 550025, China

**Keywords:** *Aletris spicata* (Thunb.) Franch., asthma, oxidative stress, inflammation, gut microbiota

## Abstract

*Aletris spicata* (Thunb.) Franch. (AS), a traditional edible and medicinal plant for treating asthma, was investigated for its therapeutic mechanisms. Liquid chromatography-high-resolution tandem mass spectrometry (LC-HRMS/MS) analysis identified 33 compounds in AS. In ovalbumin (OVA)-induced asthmatic mice, AS significantly reduced inflammatory cells (neutrophils, lymphocytes, eosinophils) in bronchoalveolar lavage fluid (BALF) and decreased IL-4, IL-5, IL-13, TNF-α, and serum IgE while increasing IFN-γ. AS alleviated lung and intestinal inflammation, reduced ROS and MDA levels, and enhanced SOD activity. Immunohistochemistry and Western blot revealed AS upregulated Nrf2/HO-1 expression and inhibited NF-κB p65 nuclear translocation. Gut microbiota studies demonstrated AS restored intestinal flora homeostasis by modulating the richness, diversity, and composition. Spearman correlation analysis identified significant relationships between oxidative stress markers, inflammatory cytokines, and specific gut bacteria. These findings indicate that AS mitigates asthma through antioxidant effects (Nrf2/HO-1 pathway), anti-inflammatory actions (NF-κB pathway), and gut microbiota modulation. The study provides a scientific basis for developing AS as a natural anti-asthma treatment or functional food. The multi-target mechanism involving oxidative stress, inflammation, and gut flora highlights AS’s comprehensive therapeutic potential for asthma management.

## Simple Summary

*Aletris spicata* (Thunb.) Franch. (AS), belonging to the Nartheciaceae family, exhibits therapeutic efficacy against asthma. However, the underlying mechanism is still unclear. In this study, the chemical composition of AS was qualitatively identified by liquid chromatography-high-resolution tandem mass spectrometry (LC-HRMS/MS). We used an ovalbumin (OVA)-induced asthma mouse model to evaluate the therapeutic effect of AS on asthma. The effects of AS on airway inflammation, oxidative stress, and gut microbiota in asthmatic mice were examined. The results showed that 33 compounds were identified from AS. It could reduce the number of inflammatory cells in bronchoalveolar lavage fluid (BALF) and the concentrations of inflammatory cytokines in BALF and serum in asthma mice. The level of oxidative stress in the lung tissue of mice was significantly reduced, and the inflammatory response in lung tissue and intestinal tissue was weakened. Moreover, AS can reduce oxidative stress and inflammatory response partly through the modulation of Nrf2 and NF-κB signaling pathways. In addition, we found that AS could also maintain the homeostasis of gut microbiota in asthma mice. These findings provide a basis for the further investigation of the potential of AS for the treatment of asthma.

## 1. Introduction

Asthma is triggered by a variety of cytokines (such as IL-4, IL-5, IFN-γ, etc.) and inflammatory medium chronic inflammatory disease of the airway [1]. It affects approximately 262 million individuals in 2019 according to survey statistics data [2]. Notably, the global incidence of this respiratory condition continues to exhibit a persistent upward trend. Furthermore, asthma claims the lives of approximately 1000 people each day and stands as the second most common chronic respiratory disease globally [3]. Characterized by heterogeneous respiratory symptoms ranging from wheezing to breathlessness and chest discomfort, asthma exhibits marked variations in clinical presentation that often result in significant life quality impairment [4]. Currently, the most common and effective treatment for asthma is inhaled corticosteroids, which can control and alleviate the attack of asthma to a certain extent, but prolonged use will lead to drug resistance and a range of adverse reactions. Consequently, the development of effective, safe, and non-toxic anti-asthmatic agents, particularly edible plant-derived medications, has emerged as a crucial research focus in asthma management [5].

The hallmark pathophysiological features of asthma comprise airway inflammation, bronchial hyperresponsiveness, allergen-specific IgE production, and mucosal hypersecretion [6]. While the exact etiological mechanisms of asthma are not fully elucidated, accumulating research demonstrates that Th1/Th2 immune imbalance plays a pivotal role in both disease initiation and clinical progression [7]. When allergens invade the body, Th2 cells proliferate and produce an abundance of cytokines, which suppress the release of Th1 cells, resulting in an imbalance of Th1/Th2 ratio [8]. As a result, a large number of inflammatory mediators are released, and then goblet cells proliferate, resulting in increased airway mucus secretion, airway thickening, airway contraction, and inducing asthma [9].

Oxidative stress is a crucial driver of asthma pathogenesis, triggering inflammation through reactive oxygen species (ROS) and depleting endogenous antioxidants [10,11]. In asthmatic airways, oxidative stress activates transcription factors that upregulate pro-inflammatory genes, leading to airway inflammation and remodeling [12]. The Nrf2 pathway provides cellular protection by dissociating from Keap1 when activated, then translocating to the nucleus to induce antioxidant genes like HO-1 [13,14]. In contrast, NF-κB acts as the master inflammatory regulator, activating in response to pathogens and cytokines to promote expression of IL-4, IL-13, and TNF-α [15,16]. Under normal conditions, Nrf2 and NF-κB maintain homeostasis through balanced signaling. However, in asthma, excessive oxidative stress disrupts this equilibrium, causing pathological crosstalk between these pathways that worsens airway inflammation [17,18]. This imbalance suggests that simultaneously activating Nrf2 while inhibiting NF-κB could be an effective therapeutic strategy for asthma [19].

Moreover, an expanding corpus of research has unveiled profound associations between intestinal microbial communities and the pathogenesis of asthma, establishing the microbiota as a critical mediator in lung–intestine tissue crosstalk during asthmatic progression [20,21]. Recent investigations have further clarified that intestinal microbiomes may mitigate asthmatic inflammation by orchestrating redox homeostasis [22]. For instance, *Bacteroides*, *Bifidobacterium*, and *Faecalibacterium prausnitzii* exert therapeutic effects by producing metabolites that enhance regulatory T cell function, thereby suppressing airway inflammation, reducing eosinophil infiltration, and inhibiting excessive mucus secretion to alleviate asthmatic symptoms [23,24]. Additionally, probiotic constituents within the intestinal flora modulate both cellular and humoral immune responses, offering protective mechanisms against allergic diseases [25]. This microbiota-mediated immunoregulatory pathway thus presents a novel therapeutic target for asthma intervention, wherein the restoration of microbial ecosystem balance holds the potential to concurrently address inflammatory and oxidative stress pathways central to the disease.

In asthma treatment, Natural products demonstrate significant clinical benefits through their multi-component, multi-target, and multi-pathway synergistic mechanisms, while exhibiting minimal adverse effects [26]. *Aletris spicata* (Thunb.) Franch. (AS) is a kind of perennial herb that belongs to the Nartheciaceae family [27]. It is a traditional food and medicinal plant. In traditional Chinese folk medicine, AS has been historically employed to treat respiratory disorders, including asthma, cough, and pneumonia [28]. At present, there are few studies on the pharmacological effects and chemical components of vermicularia. It is reported that its chemical components are mainly phenylpropanoid, steroids, flavonoids, triterpenoids, and phenolic acids, but a study on the effect and mechanism of treating asthma is absent [27,29]. We postulate that *A. spicata* extract may ameliorate asthma symptoms via multi-target mechanisms encompassing oxidative stress reduction, anti-inflammatory actions, and gut microbiota modulation. To test this hypothesis, we utilized an OVA-induced asthma mouse model to evaluate the therapeutic efficacy of AS, elucidate its mechanisms of action, and assess its regulatory effects on gut microbiota composition.

## 2. Materials and Methods

### 2.1. Materials and Reagents

In this research, the AS samples were obtained from the Guizhou Province region of southwestern China. Ovalbumin (OVA) was obtained from Sigma-Aldrich. Aluminum hydroxide (Al(OH)3) was purchased from Macklin Biochemical Co., Ltd. (Shanghai, China). Dexamethasone acetate was purchased from Xinxiang Changle Pharmaceutical Co., Ltd. (Henan, China), and each tablet contains 0.75 mg of Dexamethasone acetate. Mouse TNF-α, mouse IL-4, mouse IL-5, and mouse IL-13 ELISA Kits were purchased from MultiSciences (Lianke) Biotech Co., Ltd. (Hangzhou, China). The mouse IFN-γ ELISA kit was purchased from Shanghai Aimeng Uning Biotechnology Co., Ltd. (Shanghai, China). Mouse IgE ELISA Kit was purchased from Elabscience Biotechnology Co., Ltd. (Wuhan, China). Mouse malondialdehyde (MDA), mouse superoxide dismutase (SOD), mouse Reactive Oxygen Species (ROS) ELISA kits, and BCA protein quantification kit (BNH-C-C202) were purchased from Chongqing Bonoheng Biotechnology Co., Ltd. (Chongqing, China). NF-κB p65, Nrf2, and HO-1 Polyclonal antibody were purchased from Proteintech Group Co. Ltd. (Chicago, IL, USA). β-Tubulin Antibody was purchased from Cell Signaling Technology Co. Ltd. (Danvers, MA, USA). Hematoxylin and Eosin (H&E) and Periodic Acid-Schiff (PAS) Staining Kit were purchased from Beyotime Biotechnology Co. Ltd. (Shanghai, China). HPLC-grade acetonitrile and formic acid were purchased from Thermo Fisher Scientific Co. Ltd. (Beijing, China). All other chemicals were of analytical grade.

### 2.2. Preparation of AS Extract

Following traditional Chinese herbal decoction protocols, initially a quantity of 100 g of *A. spicata* was soaked in distilled water in an amount 10 times that of itself for 30 min. Next, it was boiled for 30 min and filtered through double cotton gauze. Afterwards, the residue was mixed with 8 times its volume of distilled water, boiled for 30 min, and then filtered. The combined filtrates were then concentrated down to 50 mL, yielding the AS extract, which was utilized for further investigation.

### 2.3. Qualitative Analysis Conditions

The chemical constituents of AS were characterized by liquid chromatography–high-resolution tandem mass spectrometry (LC-HRMS/MS). The Dionex Ultimate 3000 RSLC (HPG) system (Thermo) and a Q Exactive Focus (Thermo) equipped with a HESI-II ion source were utilized for the analysis. During the experiments, a stationary phase comprising an ACE Ultracore 2.5 Super C18 column (100 mm × 2.1 mm, 2.5 µm, Phenomenex) was employed. The mobile phase consisted of 0.1% formic acid in water and 0.1% formic acid in acetonitrile, with a gradient elution program. Mass spectrometry analysis was executed with a HESI ion source, and data acquisition was implemented in dual mode (positive and negative ion modes).

### 2.4. Animals Used in the Experiment

BALB/c female mice weighing between 18 and 22 g were obtained from Spiff (Beijing) Biotechnology Co., Ltd. (Beijing, China). The mice were housed in isolation under regulated conditions at the animal facility of the Institute of Experimental Animals, with unrestricted access to both food and water. Before commencing experimental procedures, all animals were acclimated for one week under controlled laboratory conditions, following ethical guidelines authorized by the Institutional Animal Care and Use Committee at Guizhou University of Traditional Chinese Medicine.

### 2.5. The OVA-Induced Asthma Model Establishment and Treatment Protocols

**Figure 1:**
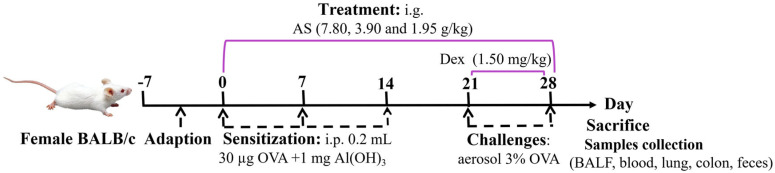
Experimental timeline of OVA–induced asthma induction and drug intervention.

A total of six groups (*n* = 7) were randomly formed among the animals. The experimental groups consisted of a normal control group, an OVA-induced asthma model group, a positive control drug group receiving Dex treatment (administered as an aqueous solution at a dosage of 1.50 mg/kg), and three experimental groups subjected to different dosages of AS. Specifically, the high-dose group was given 7.80 g/kg of AS, the medium-dose group received 3.90 g/kg, and the low-dose group was administered 1.95 g/kg (Table 1). According to the dose used in clinical practice [30] and the results of the preliminary test, this dose was the effective dose in terms of the amount of crude drug. An OVA-sensitized allergic asthma model was successfully generated using a well-characterized immunization and challenge protocol adapted from published methods and the results of our pre-experiments [31]. After a one-week adaptive feeding period, on days 0, 7, and 14, all mice received intraperitoneal injections of 0.2 mL of normal saline which contained 30 µg of OVA and 1 mg of Al(OH)_3_ for sensitization, except for those in the control group. Subsequently, from days 21 to 28, a nebulizer was used to aerosolize a 3% OVA saline challenge solution onto the mice for 30 min each time. Meanwhile, Dex was given orally to the mice 1 h before their exposure to the OVA challenge. An equivalent quantity of saline was given to the control group. Meanwhile, the treatment groups were administered different doses of AS by gavage 1 h before OVA stimulation from day 0 to day 28 (Figure 1).

### 2.6. Experimental Sample Collection

After the last excitation, all mice were transferred to freshly sterilized cages, with one per cage. Mice feces were also collected 24 h after the last dose and used for the investigation of gut microbiota. Then, all mice were anesthetized for orbital blood sampling to detect cytokines. Bronchoalveolar lavage fluid (BALF) was then additionally gathered as follows. Following proper restraint, mice were placed in a supine position on a surgical platform for tracheal exposure. A soft tube was inserted, and 300 µL of pre-cooled PBS was slowly injected, followed by suction washing. This procedure was repeated three times and a recovery rate of >80% was considered acceptable, and finally the three washes were combined to detect the count of inflammatory cells and the levels of cytokines. Subsequently, the colon tissues of the mouse were excised and rinsed thoroughly with phosphate-buffered saline (PBS). After that, they were fixed by immersion in 4% formaldehyde solution at room temperature for histopathological examination. Finally, the right lung tissue was immersed in a formaldehyde solution for histopathological and immunohistochemical analyses. Concurrently, the left lung tissue was rapidly cryopreserved in liquid nitrogen and subsequently stored at −80 °C for the quantification of oxidative stress parameters and Western blotting analysis (Figure S1).

### 2.7. Classification and Counting of Inflammatory Cells in BALF

BALF samples were processed immediately after collection through centrifugation (1500 rpm, 4 °C, 15 min). The cellular pellet obtained was reconstituted in 20 μL of PBS and gently homogenized. Aliquots (2 μL) were then transferred onto glass slides for H&E staining. Stained preparations were subsequently examined under light microscopy for cellular differentiation, quantitative analysis, and digital image acquisition.

### 2.8. Measurement of Cytokine Levels in Serum and BALF

Orbital blood specimens were subjected to centrifugation at a speed of 2500 rpm for 20 min to separate the serum. Serum total IgE and IFN-γ levels, along with BALF supernatant concentrations of IL-4, IL-5, IL-13, and TNF-α, were quantified using commercial ELISA kits following the manufacturer’s instructions.

### 2.9. Histopathological Analysis

To assess the progression of the disease as well as the effects on the intestine, the histological analyses of lung and colon tissues were performed separately. The fixed tissues of the mouse lung and colon were first dehydrated, then paraffin-embedded, and sectioned with a thickness of 4 μm. Following standard protocols, tissue sections were stained with H&E and periodic acid-Schiff (PAS). Histological evaluation was performed using a light microscope (Nikon Eclipse E100, Nikon, Tokyo, Japan), with digital images captured via an imaging system (NIKON DS-U3, Nikon, Tokyo, Japan).

### 2.10. Determination of Oxidative Stress Levels in Lung Tissue

The left lung tissue specimens of the mice were collected and then homogenized through the RIPA cold lysis approach for 3 min. Then, the centrifugal process and the supernatant of the homogenate were retrieved to measure the activity degrees of ROS, MDA, and SOD in the lung homogenate as per the guidelines provided by the kit.

### 2.11. Immunohistochemical Analysis

The lung sections were kept in an oven at a temperature of 60 °C for 2 h, subsequently dewaxed using xylene, dehydrated through a series of alcohol concentrations, placed in an extractive buffer for incubation, and finally subjected to a high-power microwave for 15 min. Following cooling and washing, slices were incubated sequentially with a range of antibodies. These comprised primary antibodies such as Nrf2 (1:200, CST, USA), HO-1 (1:200, CST, USA), and NF-κB p65 (1:200, Santa Cruz, CA, USA) along with a secondary antibody (1:2000, CST, USA) for enhanced detection purposes. Eventually, the sections underwent diaminobenzidine (DAB) staining. Then, hematoxylin reversal staining was carried out, followed by clearing using xylene and then fixation. Afterward, the glass slides were examined and snapshots were captured with the aid of a microscope (Nikon E100, Japan) and an imaging system (Nikon DS-U3, Japan). Subsequently, the Image-Pro Plus software (version 6.0) was employed to quantify the obtained images.

### 2.12. Western Blot Analysis

Total protein was extracted from the left lung tissue using manufacturer-recommended protocols for each respective kit. An equal amount of protein (30 µg) was electrophoresed in 10% SDS-PAGE at 90 V at constant pressure to cut the target band. The membrane was transferred at 300 mA at a low temperature. The cells were subjected to a 2 h incubation at room temperature with a blocking solution containing skim milk powder. The NF-κB p65 antibody, Nrf2 antibody, and HO-1 antibody were added, respectively, the membranes were removed overnight at 4 °C, and washed thoroughly in TBST. After washing the membranes, the corresponding secondary antibodies labeled with HRP (1:1000) were introduced and allowed to incubate at room temperature for an hour. Following removal and the TBST washing of the membranes, they were visualized with ECL reagents and analyzed using ImageJ software (v 1.54).

### 2.13. 16S rRNA Sequencing Analysis of Gut Microbiota

Fecal DNA samples were collected from experimental mouse groups, and the gut microbiota composition was characterized by high-throughput 16S rRNA gene sequencing on an Illumina platform. Following the manufacturer’s instructions, microbial genomic DNA was extracted and purified from each sample using a DNA extraction kit. The detailed procedure was conducted according to the reference. The V3–V4 region of the bacterial 16S rRNA gene in the genomic DNA of microorganisms was amplified through primers 341F (CCTAYGGGRBGCASCAG) and 806R (GGACTACNNGGGTATCTAAT). PCR products of equal molar concentrations were then purified, quantified, and sequenced on an Illumina Hiseq 2500 platform (San Diego, CA, USA) according to standard protocols provided by Novosource Technologies. Bioinformatics analyses were conducted using the NovoMagic platform, including the Alpha diversity index difference analysis using the Kruskal–Wallis rank sum test method and PCoA analysis using the weighted UniFrac distance method. The differential microbiota among each group were analyzed using LEfSe. Linear discriminant analysis (LDA) > 4 was considered statistically significant. Furthermore, the Spearman’s rank correlation analysis was used to analyze the correlation between the composition of the intestinal microbiota and the cytokine levels at the family and genus levels.

### 2.14. Statistical Analysis

All experiments were performed in triplicate, with results expressed as mean ± SD. Statistical analyses were conducted using GraphPad Prism software (version 9.5.1), including one-way analysis of variance (ANOVA) for intergroup comparisons. Statistical significance was defined as *p* < 0.05, and “#” was used to denote significant differences compared to the control group or the model group.

## 3. Results

### 3.1. Compositional Analysis of AS

**Figure 2:**
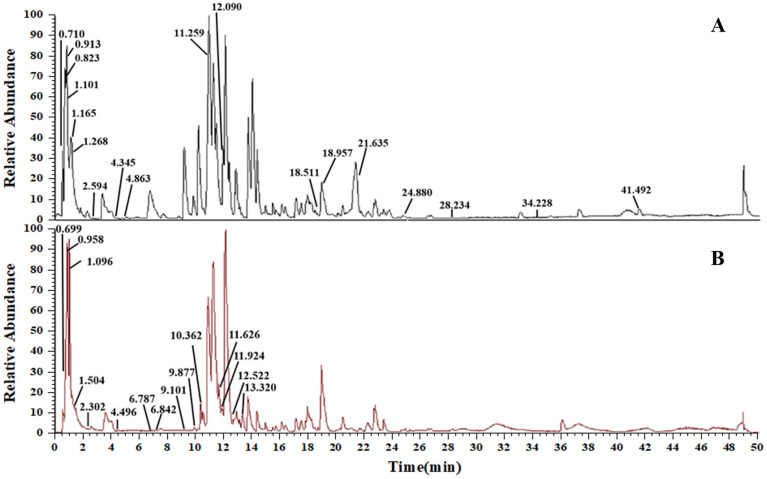
TIC diagram of AS. (**A**) Positive electrospray ionization (ESI+) mode. (**B**) Negative electrospray ionization (ESI−) mode.

By analyzing high-resolution mass spectrometer data, utilizing information from the database (Thermo Scientific Compound Discoverer 3.2, USA) and pertinent literature, thirty-three chemical components in the AS extract were deduced. Among these, 18 compounds were confirmed in positive ion mode (Figure 2A), and 15 were confirmed in negative ion mode (Figure 2B). These compounds encompassed 6 flavonoids, 5 alkaloids, 2 phenylpropanoids, 2 steroids, 10 organic acids, 2 amino acids, 1 amide, and 5 other compounds. All the identified compounds, together with their elution time, chemical name, and ionic modes are shown (Table 2).

### 3.2. Effect of AS on Inflammatory Cell Counts in BALF of Asthma Mice

**Figure 3:**
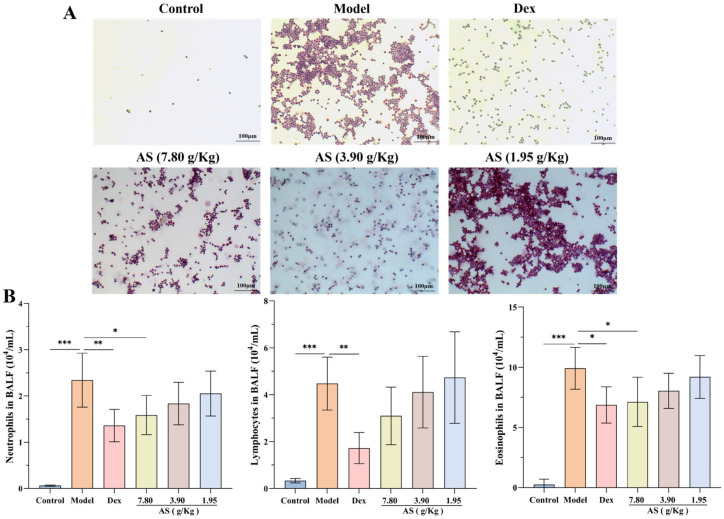
Effects of AS on BALF inflammatory cells in OVA-challenged mice. (**A**) Representative H&E staining of BAL inflammatory cells (200× magnification; scale bar: 100 µm). (**B**) Quantification of BALF inflammatory cells (neutrophils, lymphocytes, eosinophils). The data are presented as mean ± SD (*n* = 7). * *p* < 0.05, ** *p* < 0.01, *** *p* < 0.001.

Following OVA stimulation, a marked increase in the count of neutrophils, lymphocytes, and eosinophils within the BALF of the model group was observed (Figure 3A,B). Conversely, after the administration of Dex and the high dose of AS, a significant reduction in the number of inflammatory cells in the BALF was noted, with neutrophils and eosinophils showing a particularly pronounced decrease (*p* < 0.05). Even though there was no statistically significant disparity, the quantity of lymphocytes was also conspicuously lower when compared with that in the model group. These results indicate that AS lessens the infiltration of inflammatory cells within the lungs of mice suffering from asthma.

### 3.3. Effect of AS on Inflammatory Cytokine and IgE Levels of Asthma Mice

**Figure 4:**
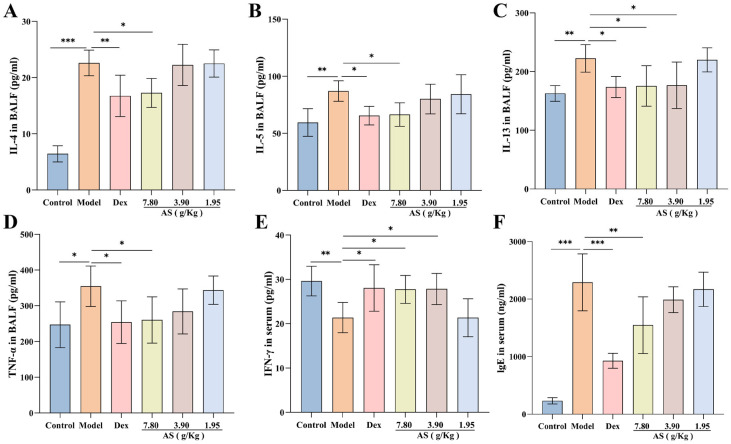
Effects of AS on cytokines in asthma mice. (**A**–**E**) Levels of BALF inflammatory cytokines (IL-4, IL-5, IL-13, TNF-α) and serum IFN-γ. (**F**) Serum immunoglobulin E (IgE) concentration. The data are presented as mean ± SD (*n* = 7). * *p* < 0.05, ** *p* < 0.01, *** *p* < 0.001.

The findings of the research revealed that, in contrast to the control group, OVA sensitization and challenge led to a substantial elevation in the production of IL-4, IL-5, IL-13, and TNF-α, with the increase in IL-4 levels being the most prominent (*p* < 0.001). Nevertheless, when compared with the model group, following the treatment with Dex and AS, particularly the high dose of AS (7.80 g/kg), these cytokine levels were markedly decreased in BALF (Figure 4A–D). Simultaneously, The serum IFN-γ levels in the model group showed a significant reduction compared to the control group (Figure 4E). In comparison to the model group, the levels in the Dex group and the high and medium doses of AS (7.80 g/kg, 3.90 g/kg) groups were significantly augmented.

As illustrated in Figure 4F, a significant elevation in the serum IgE level was observed in the model group. In comparison to the model group, a notable reduction in the level of immunoglobulin IgE was detected in both the Dex group and the high-dose AS group (*p* < 0.01). These outcomes imply that Dex and AS are capable of suppressing the overproduction of relevant cytokines in asthma, thus suppressing airway inflammation.

### 3.4. Effect of AS on Pathological Changes in Lung and Colon Tissues of Asthma Mice

**Figure 5:**
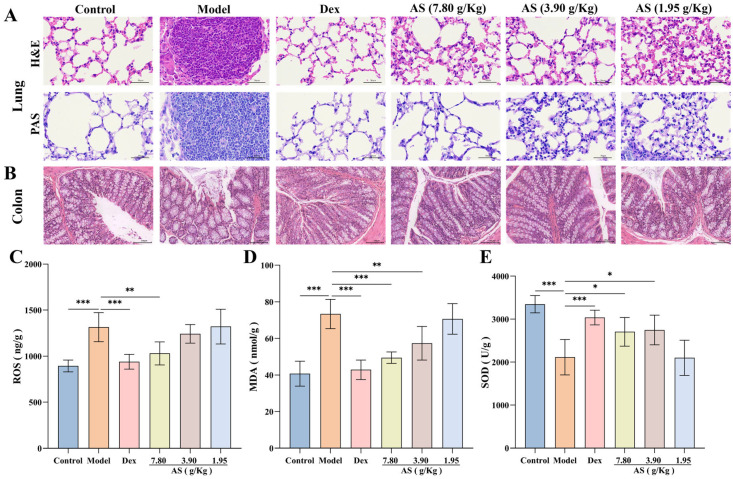
Influences of AS on the lung, colon tissues, and the levels of oxidative stress. (**A**) H&E and PAS staining of lung tissue (400× magnification; scale bar: 50 µm). (**B**) H&E staining of colon tissue (200× magnification; scale bar: 100 µm). (**C**–**E**) The concentrations of ROS, MDA, and SOD in the lung. The data are presented as mean ± SD (*n* = 3~7). * *p* < 0.05, ** *p* < 0.01, *** *p* < 0.001.

In this experiment, histopathological examination revealed significant pulmonary alterations among experimental groups (Figure 5A). Lung tissues from OVA-challenged model group mice displayed marked peribronchial inflammatory cell infiltration, along with pronounced goblet cell hyperplasia and mucus hypersecretion as evidenced by H&E and PAS staining. Therapeutic intervention with either Dex or AS significantly ameliorated these pathological features in a dose-dependent manner, with high-dose AS showing comparable efficacy to Dex in reducing airway inflammation and mucus production.

Moreover, the results of H&E staining of colonic tissues demonstrated that the colonic tissues of the mice in the control group were well-defined, structurally integral, and free from inflammatory cell infiltration (Figure 5B). In comparison to the control group, the colonic tissues of the model group mice were congested and edematous, with some of the villi in the mucosal layer detached, and inflammatory cell infiltration was evident in the mucosa and submucosa. Although the congestion and villi detachment of the colon tissue in the Dex group and AS (7.80 and 3.90 g/kg) groups were mitigated compared with the model group, a small quantity of inflammatory cell infiltration remained observable.

### 3.5. Effect of AS on Oxidative Stress Levels of Asthma Mice

The quantitative analysis revealed markedly elevated levels of ROS and MDA in the model group compared to controls, indicating substantial oxidative stress induction (Figure 5C,D). Upon Dex and AS intervention, the levels of ROS and MDA in the lung tissue of asthmatic mice were notably diminished, particularly in the Dex group and the high-dose AS group (*p* < 0.01). In contrast to the control group, the model group exhibited reduced SOD levels (Figure 5E). Notably, treatment with both Dex and AS significantly elevated SOD levels compared to the model group. Therefore, we can conclude that AS may prevent or treat asthma by reducing oxidative damage and improving an oxidative stress response in asthma mice.

### 3.6. Effect of AS on Asthma Mice via the Regulation of the Nrf2/NF-κB Signaling Pathway

**Figure 6:**
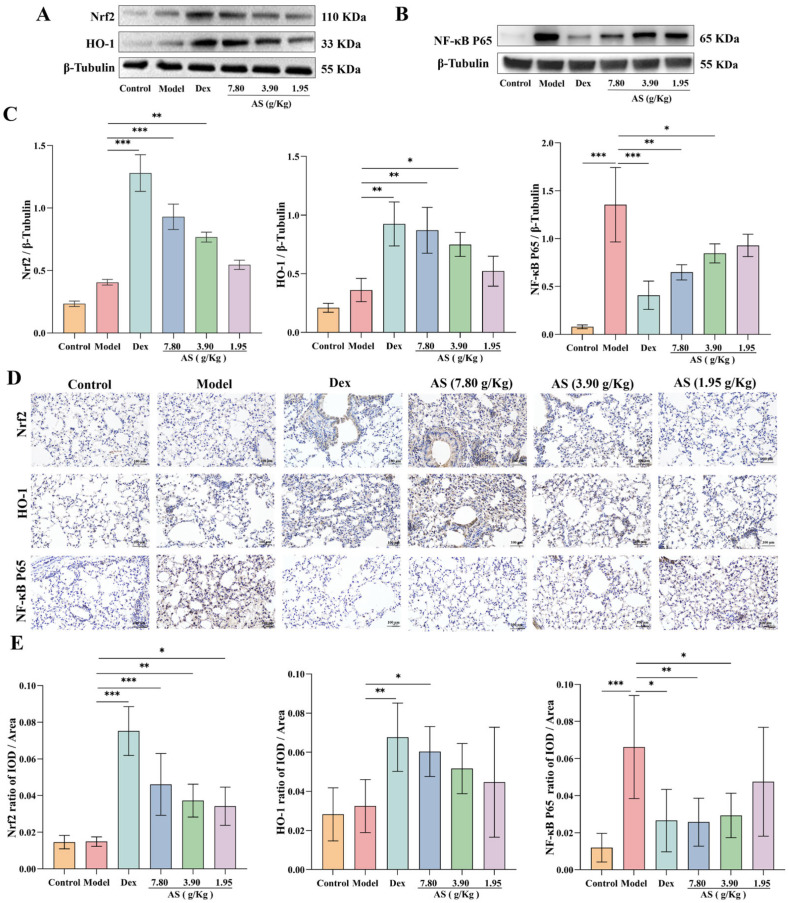
AS regulation of Nrf2/NF-κB signaling pathways. (**A**) Western blot analysis of Nrf2 and HO-1 expression. (**B**) Western blot analysis of NF-κB p65 expression. (**C**) Quantitative analysis of Nrf2, HO-1, and NF-κB p65 protein expression. (**D**,**E**) Immunohistochemical staining of Nrf2, HO-1, and NF-κB p65 (200×; Scale bar: 100 μm). The data are presented as mean ± SD (*n* = 3). * *p* < 0.05, ** *p* < 0.01, *** *p* < 0.001.

Western blot analysis demonstrated that both Dex and AS treatment significantly enhanced Nrf2 nuclear translocation and HO-1 protein expression in lung tissues compared to the model group (Figure 6A,C). The immunohistochemical results of mouse lung samples also confirmed the conclusion (Figure 6D,E). As illustrated in Figure 6, the NF-κB p65 protein level in the model group was significantly higher than that in the control group (*p* < 0.001), indicating that an inflammatory response occurred in the model group. However, after the treatment with Dex and AS (7.80 g/kg and 3.90 g/kg), the expression of NF-κB p65 in the nucleus decreased significantly (Figure 6B,C, *p* < 0.05). Furthermore, the immunohistochemical results further indicated that AS could inhibit the nuclear translocation of NF-κB p65. This indicates that AS possesses anti-oxidative stress and anti-inflammatory properties.

### 3.7. Effect of AS on Gut Microbiota of Asthma Mice

#### 3.7.1. Effect of AS on Richness and Diversity of Gut Microbiota in Asthma Mice

**Figure 7:**
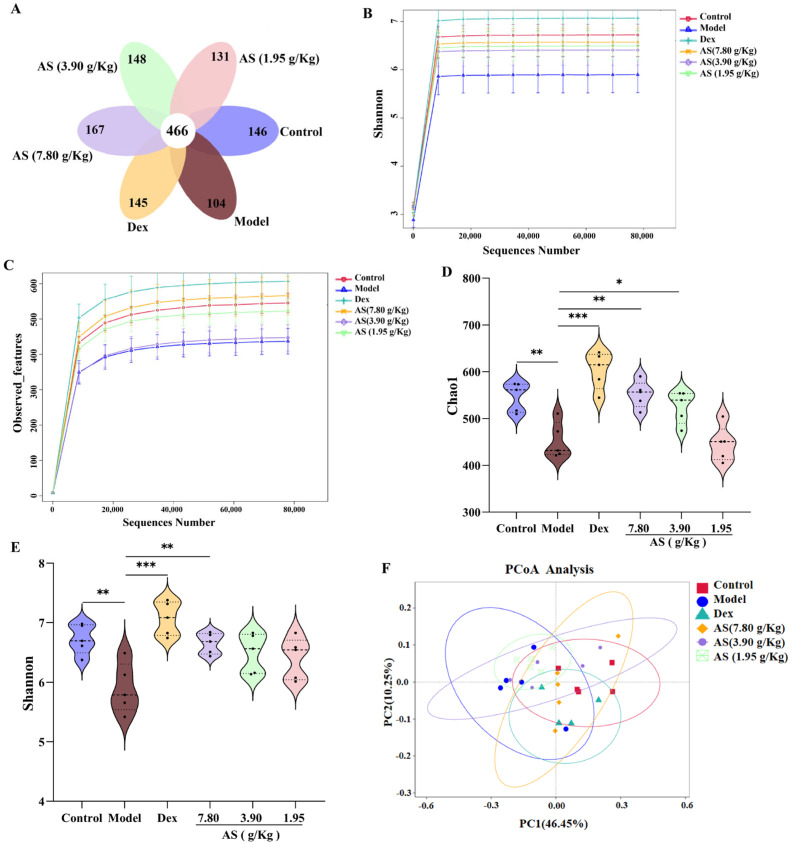
AS modulates the alterations of gut microbiota in mice with asthma. (**A**) Venn diagram. (**B**,**C**) Observed_features and Shannon curves difference group. (**D**,**E**) Analysis of the α–diversity of the gut microbiota by the Chao1 and Shannon index. (**F**) PCoA analysis. The data are presented as mean ± SD (*n* = 5). * *p* < 0.05, ** *p* < 0.01, *** *p* < 0.001.

As depicted in the Venn diagram (Figure 7A), the quantity of mutual amplicon sequence variants (ASVs) for all samples amounted to 466. In the control and model groups, there were 146 and 104 exclusive ASVs, respectively. This outcome demonstrates a substantial distinction in the microbial composition when contrasting the control mice with the asthmatic ones. Conversely, following treatment, the Dex group and each AS dose group exhibited a remarkable augmentation in microbial species in comparison to the model group. The findings from the Observed_features and Shannon curves indicated that, as the volume of sequencing data grew, the curves tended to level off, suggesting that the sequencing data was extensive enough to capture the preponderant part of the microbial information within the samples (Figure 7B,C). Subsequently, alpha diversity metrics were employed to appraise the richness and diversity of the gut microbiota. Chao1 was utilized to signify species richness, where a larger value corresponded to higher richness. Shannon was adopted to depict species diversity, with greater values denoting more pronounced diversity in the community. The results showed that the Chao1 and Shannon indices in the model group were significantly lower than those in the control group (*p* < 0.01), signifying that the richness and diversity of intestinal microorganisms in asthmatic mice had diminished (Figure 7D,E). Meanwhile, both Dex and AS treatments significantly attenuated the OVA-induced reduction in gut microbial richness and diversity, with the most pronounced effects observed in the Dex and high-dose AS (*p* < 0.01) groups compared to the model group.

#### 3.7.2. Effect of AS on Composition of Gut Microbiota in Asthma Mice

The PCoA analysis exhibited a remarkable disparity between the control and model samples (Figure 7F). In comparison with the model group, the samples from the Dex group and the AS at a dosage of 7.80 g/kg also presented evident dissimilarities and were more proximate to the samples from the control group. These findings suggest that both Dex and AS are capable of reversing the structural alterations of the intestinal microbiota in asthma mice.

**Figure 8:**
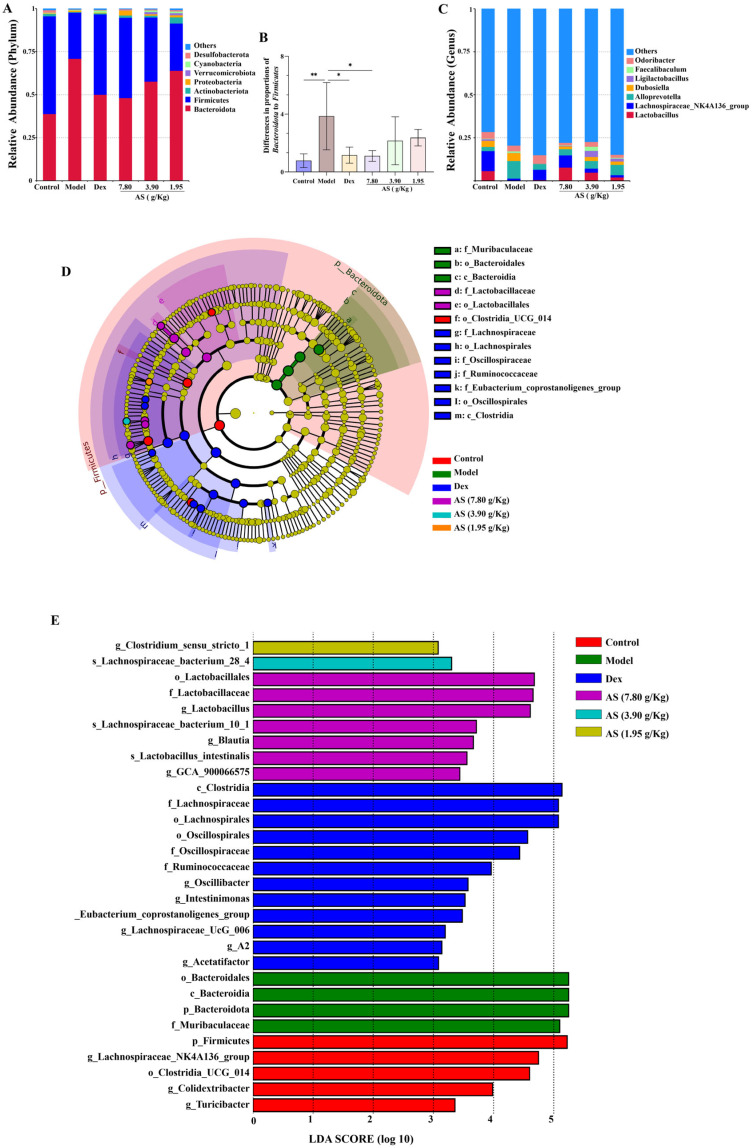
Impacts of AS on the composition of mouse intestinal microbiota. (**A**) Analysis of the community composition at the phylum level. (**B**) The ratio of *Bacteroidota* to *Firmicutes*. (**C**) Analysis of the community composition at the genus level. (**D**) Statistical chart of LEfSe analysis. (**E**) LDA discriminant result histogram. The data are presented as mean ± SD (*n* = 5). * *p* < 0.05, ** *p* < 0.01.

At the phylum level, *Bacteroidota*, *Firmicutes*, *Actinobacteriota*, and *Proteobacteria* collectively represented the predominant microbial taxa across all experimental groups. This is consistent with the findings of gut microbiota analysis reported in asthmatic patients [32]. In contrast to the model group, the relative abundance of *Bacteroidota* decreased in each treatment group, while the abundances of *Firmicutes*, *Actinobacteriota*, and *Proteobacteria* increased (Figure 8A). Meanwhile, the ratio of *Bacteroidota* to *Firmicutes* in the model group was significantly higher than that in the control group (*p* < 0.01) (Figure 8B). At the genus level, *Lactobacillus*, *Lachnospiraceae NK4A136_group*, *AlloPrevotella*, and *Dubosiella* were the representative types (Figure 8C). It was also clear that the model group increased the abundance of *AlloPrevotella* and reduced the abundances of *Lactobacillus*, *Lachnospiraceae NK4A136_group*, and *Dubosiella*, and each treatment group reversed these changes.

The results of LEfSe and LDA (Figure 8D,E) suggest that, in the control group, the contributions of *p-Firmicutes*, *g-Lachnospiraceae_NK4A136_group*, and *o_Lostridia-UCG_014* were more prominent; in the model group, the influence of *p_Bacteroidota* and *f_Muribaculaceae* was stronger; and in the AS (3.90 g/kg) group, the influence of *g_Lactobacillus* was greater. Collectively, these results suggest that AS is capable of modifying the gut microbiota composition in asthmatic mice.

#### 3.7.3. Correlation Analysis of Oxidative Stress Levels, Cytokines, and Gut Microbiota

**Figure 9:**
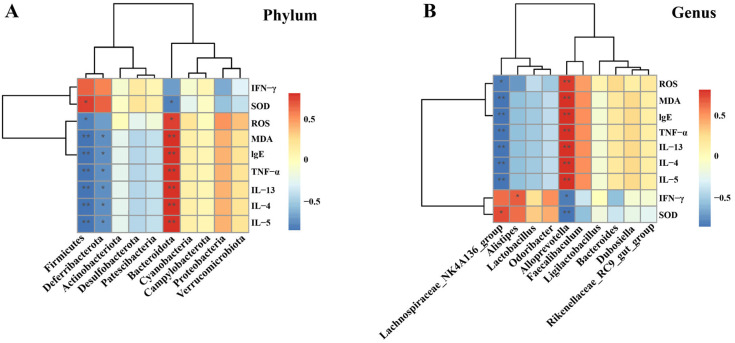
Correlation analysis between cytokines, oxidative stress levels, and microbiota. (**A**) At the phylum level; and (**B**) At the genus level. The data are presented as the mean ± SD (*n* = 5). * *p* < 0.05, ** *p* < 0.01.

The Spearman’s rank correlation analysis at the Phylum level is illustrated (Figure 9A). It was revealed that a total of 3 microbial communities exhibited significant correlations with oxidative stress levels and multiple cytokines. Specifically, *Firmicutes* had a positive correlation with SOD while showing a negative correlation with ROS, MDA, IgE, TNF-α, IL-13, IL-4, and IL-5. *Deferribacterota* was negatively correlated with MDA, IgE, TNF-α, IL-13, IL-4, and IL-5. *Bacteroidota* was negatively correlated with SOD and positively correlated with ROS, MDA, IgE, TNF-α, IL-13, IL-4, and IL-5. Further analysis was conducted at the genus level (Figure 9B). The results indicated that the *Lachnospiraceae_NK4A136_group* was negatively correlated with ROS, MDA, IgE, TNF-α, IL-13, IL-4, and IL-5, and positively correlated with SOD. *Alistipes* was positively correlated with IFN-γ. *Alloprevotella* was positively correlated with ROS, MDA, IgE, TNF-α, IL-13, IL-4, and IL-5 (*p* < 0.01), and negatively correlated with IFN-γ and SOD.

## 4. Discussion

Currently, asthma impacts approximately 4.3% of the global population, imposing a substantial burden on governments, healthcare systems, families, and patients alike [2]. Asthma pathogenesis involves complex interactions between genetic predisposition, environmental triggers, and immune system dysregulation [33]. Notably, the excessive release of inflammatory mediators, stemming from the induction of oxidative stress and augmented pulmonary inflammatory cell infiltration, represents crucial aspects of its pathogenesis [34]. Meanwhile, a growing body of research has indicated that alterations in the intestinal flora are also intimately tied to the onset, progression, and treatment of asthma [35]. Given the extensive sources, minimal side effects, diverse chemical architectures, and multi-target synergistic capabilities of plant medicine, the exploration of asthma medications derived from natural products might offer a novel avenue and strategy [36]. AS is predominantly found in China, Japan, Malaysia, and the Philippines [37]. In addition to being used in vegetable consumption, AS is also used as a medicine for asthma among people in China, but its exact mechanism of action remains unclear [28]. Consequently, in this particular study, a comprehensive pharmacological methodology was employed to examine the influence of AS on OVA-induced asthma in mice, as well as its impact on the gut microbiota, to uncover the underlying molecular mechanisms. The findings of this study revealed that notable pathological alterations were detected in the lung tissues of mice in the model group. There was a substantial influx of inflammatory cells encircling the bronchi within the lung tissue, accompanied by elevated mucus secretion and a marked upsurge in the serum IgE level. These findings signified the successful establishment of the model, which served as the foundation for all ensuing experiments.

The fundamental constituents of herbal medicine constitute the crux of drug research and new drug development endeavors. In the present experiment, LC-HRMS/MS was employed to identify and scrutinize the pertinent chemical components within AS. A total of thirty-three compounds were discerned in AS, encompassing flavonoids, alkaloids, and phenylpropanoids. Prior investigations have demonstrated that the total flavonoids of AS possess certain antioxidant and anti-inflammatory capabilities, which might be correlated with the curative effect of AS on asthma [38]. Moreover, it has been documented in the literature that a variety of chemical components in AS play a therapeutic role in asthma. For instance, the flavonoid vitexin is capable of reducing the levels of Th2 cytokines in OVA-induced asthma mouse models, alleviating inflammatory cell infiltration in lung tissue, and suppressing the expression of NF-κB protein [39]. The alkaloid compound Betaine can ameliorate airway inflammation in the lung tissue of OVA-induced asthma mouse models and augment the content of MDA, a marker of lipid peroxidation, which may be associated with the antioxidant activity of betaine [40]. Caffeic acid, a phenylpropanoid compound, can attenuate inflammation in lung tissue and reduce the levels of Th2 cytokines [41]. The aforementioned chemicals, similarly to AS, can exert certain therapeutic impacts on asthma mice and are related to anti-inflammatory and antioxidant stress, potentially serving as the therapeutic substances of AS in the treatment of asthma.

Asthma is a complex disease involving multiple cell types (such as T lymphocytes and macrophages) and cytokines, with the activation of these cellular components triggering asthmatic responses [42]. The pathogenic mechanisms of each cell vary [43]. Among these, T lymphocytes exert an immunomodulatory effect in the pathogenesis of asthma [44]. Recent studies have proposed that the imbalance between Th1 and Th2 subsets of helper T cells is a significant mechanism underlying asthma [45]. Th1 cells secrete cytokines such as IFN-γ, TNF-α, and IL-12, which primarily mediate the immune responses associated with local inflammation and are involved in cellular immunity and the development of delayed hypersensitivity inflammation [46]. Despite their common Th1 lineage, IFN-γ and TNF-α exhibit divergent immunomodulatory effects in inflammatory pathologies [47]. Specifically, TNF-α can indirectly drive inflammatory responses by initiating inflammatory immune responses and promoting disease progression [48]. Th2 cells mainly secrete cytokines including IL-4, IL-5, and IL-13 to stimulate the occurrence of type Ⅱ immune responses. The activation of Th2 cells leads to the production of interleukins, which further activates B lymphocytes and enables them to synthesize specific IgE [49]. Allergen-specific IgE binds to high-affinity receptors on mast cells and basophils, triggering degranulation and subsequent inflammatory cascade activation [50]. Th1 and Th2 cells exhibit mutual antagonism, with their functional equilibrium maintained through the reciprocal inhibition of cytokine production and effector mechanisms [51]. In the experiment, after OVA stimulation, the levels of IL-4, IL-5, IL-13, and TNF-α in BALF and the level of IgE in the serum of mice in the asthma model group were significantly elevated, indicating that Th2 cells were overly active. Serum IFN-γ levels were significantly reduced in the model group compared to the controls, indicating Th1/Th2 immune imbalance. This imbalance causes an abnormal immune response and subsequently leads to the occurrence of asthma. Meanwhile, histopathological examination revealed characteristic inflammatory infiltrates in both the pulmonary and colonic tissues of asthmatic mice. After treatment with AS, particularly in the AS (7.80 g/kg) group, the inflammatory infiltration in the lung and colon tissues was significantly alleviated, and the levels of TNF-α in BALF and IgE in the serum were decreased. At the same time, the Th1/Th2 imbalance induced by OVA stimulation was reversed by reducing the Th2 cytokines (IL-4, IL-13) and increasing Th1 cytokines like IFN-γ, thereby reducing airway inflammation and improving the airway remodeling in mice. Collectively, these findings demonstrate the therapeutic potential of AS in ameliorating asthma pathology.

More and more studies have found that oxidative stress has become a major factor in the pathogenesis of asthma [52]. Studies have demonstrated that excessive ROS generated by diverse cell types in the airways of asthma patients will give rise to a greater quantity of oxidative stress substances during asthma episodes. This, in turn, augments the infiltration of inflammatory cells into the lungs and prompts the elevation of proinflammatory cytokines in the airways [53]. MDA represents one of the by-products of the peroxidation of unsaturated fatty acids within cells and is also a widely acknowledged indicator of oxidative stress [54]. Disrupted ROS homeostasis depletes endogenous antioxidants including SOD and CAT, resulting in oxidative damage to cellular macromolecules and tissue injury [13]. In the current investigation, it was ascertained that AS reduced the content of ROS and MDA and enhanced the activity of SOD in lung tissues, thereby suppressing the oxidative stress elicited by OVA. The aforementioned results imply that AS is capable of alleviating OVA-induced asthma by mitigating oxidative stress.

To further clarify the AS regulatory mechanism of oxidative stress and the inflammation of asthma, we examined the impacts of AS on the Nrf2/NF-κB signaling pathways. Numerous studies have indicated that Nrf2 and NF-κB are crucial targets for asthma treatment [55]. The Nrf2/HO-1 is regarded as the primary defense mechanism against oxidative damage. Among them, HO-1 can effectively reduce airway inflammation and mucus secretion in asthma patients, while Nrf2 can alleviate the asthmatic phenotype in asthma model mice [56]. NF-κB is a protein complex that functions as a transcription factor and holds a pivotal position in the development and progression of inflammatory diseases [57]. NF-κB pathway activation drives the transcriptional upregulation of multiple proinflammatory mediators, including TNF-α and Th2-associated cytokines (IL-4, IL-5, IL-13), which collectively perpetuate allergic airway inflammation [58,59]. Extensive studies have established bidirectional crosstalk between Nrf2 and NF-κB pathways in asthma pathogenesis, with Nrf2 activation exerting the inhibitory effects on NF-κB-driven inflammatory responses [14,60]. In addition, in asthmatic mice, OVA induction can promote the activation of the NF-κB pathway, leading to the excessive production of ROS in lung tissue, thereby causing Nrf2-related oxidative stress [61]. The results of the current study demonstrated that AS markedly enhanced the expression of Nrf2 and HO-1, and suppressed the level of NF-κB p65 in asthma mice.

Moreover, numerous research studies have pointed out that the gut microbiota plays a crucial role in the occurrence and development of asthma [62]. Based on the theory of “exterior-interior correlation between the lung and large intestine” from Huang Di Nei Jing (the Yellow Emperor’s Canon of Internal Medicine) along with modern research, it has been further verified that the lung and intestine interact both pathologically and physiologically through the microbiota [63]. The ecological imbalance of intestinal fungi can influence the risk of asthma in children and the severity of asthma in adults by regulating the immune system related to lung immunity [64]. Research has discovered that the gut microbiota of patients and animal models with asthma have undergone significant alterations compared to normal humans and animals [65]. These changes include a reduction in microbial abundance and diversity, as well as an imbalance between potentially beneficial and harmful microbiota [66,67]. Consistent with previously reported results, Alpha diversity index analysis in the present study showed a significantly reduced Chao1 and Shannon indices in the model group mice, while Weighted_Unifrac-based PCoA scores showed significant differences in microbial community composition between the model group and control group. The results showed that the species richness and diversity of intestinal flora in the model group were significantly reduced. This may be related to the destruction of the ecological environment of intestinal flora caused by OVA inhalation.

The structure of the gut microbiota at the phylum and genus levels was further analyzed, along with its correlation with cytokines and oxidative stress levels. At the phylum level, the dominant bacterial taxa in all groups were *Bacteroidota* and *Firmicutes*, which are also the primary microorganisms in the gut microbiota of mice, accounting for nearly 90% of the entire gut microbiota. This coincides with the findings of a large number of previous studies [68]. Studies have demonstrated that *Bacteroidota* is closely related to human immunity and can regulate Th1/Th2 to achieve a balance [69,70]. The relevant analysis of the experiment showed that *Bacteroidota* was positively correlated with Th2 (IL-13, IL-4, IL-5) inflammatory factors and negatively correlated with Th1 cytokines (IFN-γ). Hence, it is hypothesized that *Bacteroidota* might exert a protective effect in asthma resulting from Th1/Th2 imbalance. Previous studies have established a positive association between *Firmicutes* abundance and Th1 cell responses [71]. The results of this study found that, although there was no significant correlation between *Firmicutes* and Th1 cytokines (IFN-γ), there was a positive correlation trend. However, *Firmicutes* was negatively correlated with Th2 cytokines, yet the specific mechanism requires further exploration. Additionally, an increase in the relative abundance of *Bacteroidota* to *Firmicutes* (*B/F* ratio) has been proposed to be important in alleviating oxidative stress and asthma [45,72]. *Deferribacterota*, which has a significant association with cytokine and oxidative stress levels in the gut microbiota of asthmatic mice, plays a complex role in inflammatory diseases. It can either reduce inflammation or exacerbate it by influencing intestinal metabolites [73]. In this study, *Deferribacterota* may play a beneficial role in asthma, which needs to be further confirmed. Secondly, at the genus level, the abundances of *Lachnospiraceae_NK4A136_group* and *Alloprevotella* were significantly changed in each group, and they were significantly correlated with the levels of cytokines and oxidative stress. Studies have found that *Lachnospiraceae_NK4A136_group* is a potentially beneficial bacterium, and its metabolites can improve inflammation by inhibiting the NF-κB pathway and reducing TNF-α levels [74]. At the same time, this bacterium was positively correlated with SOD and negatively correlated with MDA content [75], which was consistent with the results of this study. The abundance of *Alloprevotella* was significantly upregulated in the colitis mice model [76], suggesting that *Alloprevotella* may be associated with inflammation and can induce or exacerbate inflammatory responses, which needs to be further verified. In addition, *Alistipes* was found to be positively correlated with IFN-γ in the leukemia mouse model related to the immune system [77], which was in line with the results of this study.

While this study demonstrates that AS confers protection against oxidative stress and inflammation in asthma through gut microbiota modulation, several limitations should be acknowledged. First, in terms of experimental design, since AS intervention was initiated during the sensitization phase, it remains unclear whether its effects primarily influence immune regulation in the sensitization phase, inflammatory responses in the challenge phase, or act throughout both stages. Stage-specific dosing experiments (e.g., administering AS only during the sensitization phase or only during the challenge phase) are needed to clarify this, which will provide direct evidence for dissecting the critical time window of AS intervention in asthmatic immune responses. Second, at the mechanistic level, while the involvement of the Nrf2/NF-κB signaling pathway has been preliminarily confirmed, causal validation is required through gene knockout animal models or specific inhibitors to establish the necessity of this pathway. More importantly, numerous key questions regarding the specific mechanisms of gut microbiota regulation remain unresolved, including which key bacterial genera and their metabolites play dominant roles, the specific regulatory pathways of the microbiota–gut–lung axis, and the spatiotemporal association between microbiota changes and Nrf2/NF-κB pathway activation. These mechanistic investigations require systematic analysis using multi-omics technologies (such as metagenomics and metabolomics) combined with germ-free animal models. Such in-depth studies will not only refine the theoretical understanding of AS’s mechanisms but also provide precise regulatory targets for clinical translation.

## 5. Conclusions

In conclusion, this study demonstrates that AS effectively alleviates oxidative stress and inflammation in OVA-induced asthma through the modulation of the Nrf2/NF-κB signaling pathway, potentially mediated by gut microbiota restoration. These findings not only validate traditional applications of AS but also provide a modern pharmacological framework for its therapeutic effects. The demonstrated multi-target efficacy positions AS as a promising candidate for developing integrative asthma therapies that bridge phytomedicine and microbiome modulation.

## Figures and Tables

**Table 1:** Animal grouping and treatment protocol.

Group	Adaption	Sensitization	Challenges	Treatment
		Day 0, 7, 14	Day 21–28	
Control	Normal feeding	i.p. 0.2 mL saline	Aerosol saline (30 min/day)	Day 0–28 i.g. 0.2 mL saline, once daily
Model	Normal feeding	i.p. 0.2 mL saline (30 µg OVA + 1 mg Al(OH)_3_)	Aerosol 3% OVA (30 min/day)	Day 0–28 i.g. 0.2 mL saline, once daily
Dex (1.50 mg/kg)	Normal feeding	Same as OVA model	Same as OVA model	Day 21–28 i.g. 0.2 mL Dex (1.50 mg/kg), once daily
AS (7.80 g/kg)	Normal feeding	Same as OVA model	Same as OVA model	Day 0–28 i.g. 0.2 mL AS (7.80 g/kg), once daily
AS (3.90 g/kg)	Normal feeding	Same as OVA model	Same as OVA model	Day 0–28 i.g. 0.2 mL AS (3.90 g/kg), once daily
AS (1.95 g/kg)	Normal feeding	Same as OVA model	Same as OVA model	Day 0–28 i.g. 0.2 mL AS (1.95 g/kg), once daily

**Table 2:** LC-HRMS/MS analysis of the chemical composition of AS extract.

**No.**	**Retention Time** **(min)**	**Chemical Name**	**Ion** **Mode**	**No.**	**Retention Time** **(min)**	**Chemical Name**	**Ion** **Mode**
1	0.699	D-(+)-Arabitol	[M + H]^−^	18	9.101	2.2-Dimethylglutaric acid	[M + H]^−^
2	0.710	Choline	[M + H]^+^	19	9.877	D(+)-Phenyllactic acid	[M + H]^−^
3	0.823	DL-Arginine	[M + H]^+^	20	10.362	Vicenin II	[M + H]^−^
4	0.913	Hypoxanthine	[M + H]^+^	21	11.259	Vicenin	[M + H]^+^
5	0.958	Acetyl-DL-glutamic acid	[M + H]^−^	22	11.626	Isoschaftoside	[M + H]^−^
6	1.096	3-Hydroxy-3-methylglutaric acid	[M + H]^−^	23	11.924	Schaftoside	[M + H]^−^
7	1.101	Betaine	[M + H]^+^	24	12.090	Glucosylvitexin	[M + H]^+^
8	1.165	DL-Malic acid	[M + H]^+^	25	12.522	Vitexin	[M + H]^−^
9	1.268	Guanine	[M + H]^+^	26	13.320	Azelaic acid	[M + H]^−^
10	1.504	Citric acid	[M + H]^−^	27	18.511	Testosterone cypionate	[M + H]^+^
11	2.302	Protocatechuic acid	[M + H]^−^	28	18.957	9-Oxo-10(E),12(E)-octadecadienoic acid	[M + H]^+^
12	2.594	Maltol	[M + H]^+^	29	21.635	19-Nortestosterone	[M + H]^+^
13	4.345	Acetophenone	[M + H]^+^	30	24.880	Prostaglandin B1	[M + H]^+^
14	4.496	2-Isopropylmalic acid	[M + H]^−^	31	28.234	α-Linolenic acid	[M + H]^+^
15	4.863	Ritalinic acid	[M + H]^+^	32	34.228	Hexadecanamide	[M + H]^+^
16	6.787	Caffeic acid	[M + H]^−^	33	41.492	Erucamide	[M + H]^+^
17	6.842	3-Hydroxycaproic acid	[M + H]^−^				

## Data Availability

Data will be made available upon request.

## Supplementary Materials

The following supporting information can be downloaded at https://www.mdpi.com/article/10.3390/biology14060731/s1, Figure S1: The original Western blot images of the Nrf2/NF-κB signaling pathway regulated by AS in asthmatic mice. (A) Nrf2 protein. (B) HO-1 protein. (D) NF-κB p65 protein. (C,E) β-Tubulin (loading control).

## Abbreviations

The following abbreviations are used in this manuscript:

OVA	Ovalbumin
Dex	Dexamethasone acetate
IL−4	Interleukin−4
IL−5	Interleukin−5
IL−13	Interleukin−13
TNF−α	Tumor necrosis factor α
IFN−γ	Recombinant human interferon gamma
IgE	Immunoglobulin E
MDA	Malondialdehyde
SOD	Superoxide dismutase
ROS	Reactive oxygen species
BALF	Bronchoalveolar lavage fluid
H&E	Hematoxylin and eosin
PAS	Periodic acid-schiff
NF−κB P65	Phospho nuclear factor−κB p65
Nrf2	Nuclear Factor erythroid 2–Related Factor 2
HO−1	Heme oxygenase 1
PCoA	Principal coordinates analysis
LEfSe	Linear discriminant analysis effect size
