# Supplementary material for: Mechanisms of Aletris spicata (Thunb.) Franch. Extract in Asthma Therapy: Oxidative Stress, Inflammation, and Gut Microbiota
Source: Biology (Basel). 2025 Jun 19;14(6):731. doi: 10.3390/biology14060731 (PMC12189390; doi:10.3390/biology14060731)
Supplement: Supplementary file 1 [file biology-14-00731-s001.zip › biology-3659531-supplementary.pdf]

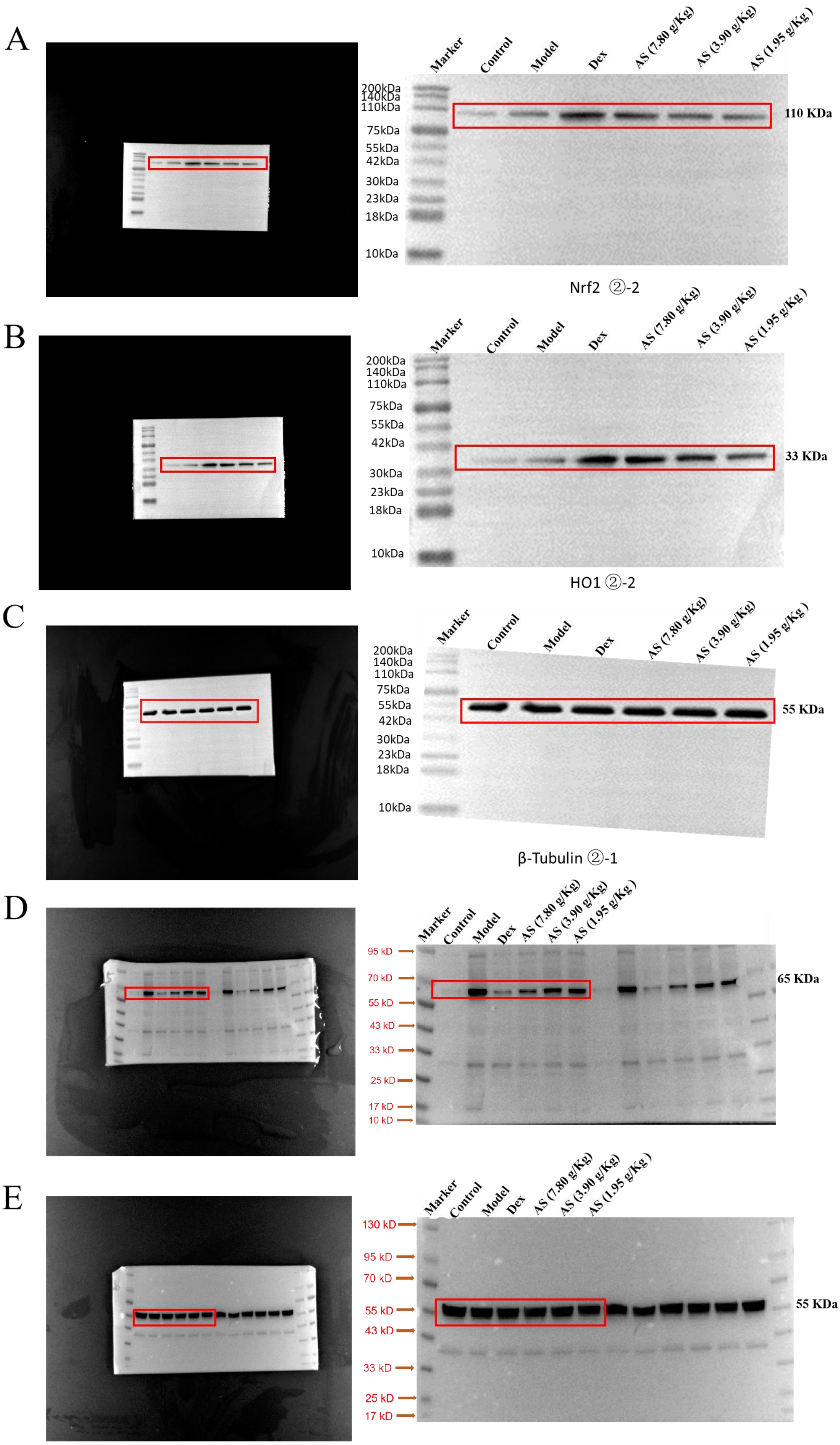
A
Marker
Control
Model
Dex
AS (7.80 g/Kg)
AS (3.90 g/Kg)
AS (1.95 g/Kg)
200kDa
140kDa
110kDa
75kDa
55kDa
42kDa
30kDa
23kDa
18kDa
10kDa
110 KDa
Nrf2 ②-2
B
Marker
Control
Model
Dex
AS (7.80 g/Kg)
AS (3.90 g/Kg)
AS (1.95 g/Kg)
200kDa
140kDa
110kDa
75kDa
55kDa
42kDa
30kDa
23kDa
18kDa
10kDa
33 KDa
HO1 ②-2
C
Marker
Control
Model
Dex
AS (7.80 g/Kg)
AS (3.90 g/Kg)
AS (1.95 g/Kg)
200kDa
140kDa
110kDa
75kDa
55kDa
42kDa
30kDa
23kDa
18kDa
10kDa
55 KDa
β-Tubulin ②-1
D
Marker
Control
Model
Dex
AS (7.80 g/Kg)
AS (3.90 g/Kg)
AS (1.95 g/Kg)
95 kD
70 kD
55 kD
43 kD
33 kD
25 kD
17 kD
10 kD
65 KDa
E
Marker
Control
Model
Dex
AS (7.80 g/Kg)
AS (3.90 g/Kg)
AS (1.95 g/Kg)
130 kD
95 kD
70 kD
55 kD
43 kD
33 kD
25 kD
17 kD
55 KDa

Figure S1. The original Western Blot images of the Nrf2/NF-κB signaling pathway regulated by AS in asthmatic mice. (A) Nrf2 protein. (B) HO-1 protein. (D) NF-κB p65 protein. (C, E) β-Tubulin (loading control).
